# Visual cue training to improve walking and turning after stroke: a study protocol for a multi-centre, single blind randomised pilot trial

**DOI:** 10.1186/1745-6215-14-276

**Published:** 2013-09-03

**Authors:** Kristen L Hollands, Trudy Pelton, Andrew Wimperis, Diane Whitham, Sue Jowett, Catherine Sackley, Wing Alan, Paulette van Vliet

**Affiliations:** 1 Research Fellow School of Health Sciences, University of Salford, Allerton Building, Salford M6 6PU, UK; 2 Colleges of Life and Environmental Sciences & Medical and Dental Sciences, University of Birmingham, Edgbaston, Birmingham B15 2TT, UK; 3 Birmingham Community Health Care NHS Trust, (BCHCT), Moseley Hall Hospital, Birmingham B13 8JL, UK; 4 University of Nottingham, Nottingham Clinical Trials Unit, Nottingham Health Science Partners, C-floor, South Block, Queen’s Medical Centre, Nottingham NG7 2UH, UK; 5 University of East Anglia, School of Allied Health Professions, Queens Building, Earlham Road, Norwich, Norfolk NR4 7TJ, UK; 6 University of Newcastle, School of Health Sciences, Newcastle, Hunter Building Callaghan, University Drive, Callaghan, NSW 2308, Australia

**Keywords:** Gait, Rehabilitation, Stroke, Vision

## Abstract

**Background:**

Visual information comprises one of the most salient sources of information used to control walking and the dependence on vision to maintain dynamic stability increases following a stroke. We hypothesize, therefore, that rehabilitation efforts incorporating visual cues may be effective in triggering recovery and adaptability of gait following stroke*.* This feasibility trial aims to estimate probable recruitment rate, effect size, treatment adherence and response to gait training with visual cues in contrast to conventional overground walking practice following stroke.

**Methods/design:**

A 3-arm, parallel group, multi-centre, single blind, randomised control feasibility trial will compare overground visual cue training (O-VCT), treadmill visual cue training (T-VCT), and usual care (UC). Participants (n = 60) will be randomly assigned to one of three treatments by a central randomisation centre using computer generated tables to allocate treatment groups. The research assessor will remain blind to allocation. Treatment, delivered by physiotherapists, will be twice weekly for 8 weeks at participating outpatient hospital sites for the O-VCT or UC and in a University setting for T-VCT participants.

Individuals with gait impairment due to stroke, with restricted community ambulation (gait speed <0.8m/s), residual lower limb paresis and who are able to take part in repetitive walking practice involving visual cues (i.e., no severe visual impairments, able to walk with minimal assistance and no comorbid medical contraindications for walking practice) will be included.

The primary outcomes concerning participant enrolment, recruitment, retention, and health and social care resource use data will be recorded over a recruitment period of 18 months. Secondary outcome measures will be undertaken before randomisation (baseline), after the eight-week intervention (outcome), and at three months (follow-up). Outcome measures will include gait speed and step length symmetry; time and steps taken to complete a 180° turn; assessment of gait adaptability (success rate in target stepping); timed up and go; Fugl-Meyer lower limb motor assessment; Berg balance scale; falls efficacy scale; SF-12; and functional ambulation category.

**Discussion:**

Participation and compliance measured by treatment logs, accrual rate, attrition, and response variation will determine sample sizes for an early phase randomised controlled trial and indicate whether a definitive late phase efficacy trial is justified.

**Trial registration:**

Clinicaltrials.gov, NCT01600391.

## Background

Recovery of walking function is a major goal of rehabilitation after stroke. Although many patients regain a basic locomotor pattern, one study has reported that only 7% of patients discharged from rehabilitation are able to walk safely in the community [1]. A further study suggests that as many as 50% of stroke patients discharged into the community will fall and that a large proportion of these falls will occur during manoeuvres in which the basic walking pattern needs to be adapted, such as in turning [2]. Recent studies have postulated that impairments in gait which persist after stroke, such as diminished speed, asymmetries in step lengths and gait phase durations, may have an underlying impoverished ability to adapt the gait pattern as required to mobilize independently [3]. Hence, the incidence of falls after stroke may be due to impaired ability to flexibly adapt an already impoverished coordination pattern during straight walking [4] in order to turn, step over an obstacle, target a safe foot-placement, or alter speed as needed for independent community ambulation [3].

Current approaches to rehabilitation of gait following stroke are varied, based on different models of motor physiology and disease recovery, but most share targeting motor impairments during straight walking only as opposed to adaptive walking ability [5]. Overall, evidence indicates that current rehabilitation approaches have only modest effects on impairment and activity [6,7]. Therefore, new more effective treatments need to be developed and tested within robust, early phase studies. Treatments should be supported by a sound theoretical basis; specifically, by an understanding of the mechanisms which cause gait deficits and proposed treatment effects [7].

Evidence from the motor learning literature indicates that effective neurorehabilitation requires task-specific practice that should be varied, intensive [7], and driven by a combination of extrinsic movement goals and implicit knowledge of movement control [8]. A recent synthesis of existing evidence further suggests that task-specific practice of walking which targets restoration of gait coordination patterns (temporal and spatial symmetry) may be beneficial in improving overall walking function [4]. The goal of normalizing walking patterns, particularly symmetry, is controversial given enduring neuromuscular asymmetries after stroke [9]. However, meta-analysis indicates that interventions that show most promise for improvements in walking function (task-specific locomotor practice and auditory cueing) both involve repetition of a more normative gait pattern, while the least beneficial (ankle-foot orthoses/functional electrical stimulation and exercise) do not explicitly practice a normative gait pattern [4]. The findings from the systematic examination of the evidence base lend support to the notion that repeated exposure to normalized movement patterns could bring about positive changes in motor control [10,11] and support the development of interventions that enable patients to undertake intensive practice of functional tasks in a manner that drives an optimised movement pattern.

Rehabilitation approaches identified as showing the most promise for eliciting normalized gait coordination patterns utilised auditory cues as extrinsic movement goals [4]. While there are good indications that stroke survivors are able to adjust gait coordination in response to auditory cues [12], some studies have shown that visual cues may be more effective in triggering gait adjustments in healthy participants walking straight [13].

Current understanding of motor control of locomotion indicates that visual information comprises one of the most important and salient sources of information used during walking [4,14] and that stroke survivors have been reported to become more dependent on vision to maintain dynamic stability [15]. Paradigms involving walking in response to visual cues have recently begun to be used to investigate functional walking tasks, including turning and obstacle avoidance, in various patient populations, both overground and on a treadmill [13,16-18]. Despite numerous small experimental studies reporting the potential efficacy of using visual cues to enhance gait function, to date, there have been no robust clinical trials of these interventions that we are aware of.

Based on the current understanding of motor control of walking and stroke rehabilitation, we hypothesize that visual cues would be more effective in triggering gait recovery and adaptability following stroke than interventions not including visual cues. It is hypothesized that anticipated improvements to functional gait may be derived from task-specific practice of regulating changes in the relation between the base of support and the centre of mass occurring when step widths and lengths change, which is crucial for dynamic balance control.

The study reported here comprises an early phase pilot randomised controlled trial (RCT) aiming to examine the feasibility of a trial comparing task-specific locomotor practice incorporating visual cues to usual care rehabilitation, which does not include visual cues. Specifically, the study aims to:

1. Characterise participants who are included and excluded into the trial from four NHS trusts in the West Midlands.

2. Provide an estimation of recruitment rates to the trial across the multiple sites.

3. Estimate the adherence of participants allocated to the visual cue training (VCT) to the prescribed dose.

4. Present the completeness of proposed outcome data.

5. Calculate sample sizes for a subsequent definitive trial, based on measured changes in performance for usual care and VCT intervention groups.

6. Determine participant tolerance of the VCT intervention.

7. Determine therapist acceptability for delivering the VCT interventions.

8. Collate health and social care resource data to inform data collection methods for an economic evaluation in the subsequent definitive trial.

## Methods

### Design

This is a pilot, multi-centre, randomised [1:1:1], stratified by gait speed (Severe group: <0.4 m/s; Moderate group: between 0.4 m/s and 0.8 m/s [19]), controlled trial with three parallel groups and single-blind assessment conducted in the UK (four sites).

### Randomisation

The randomisation will be created using StataSEv9 (StataCorp, College Station, TX, USA) statistical software with a 1:1:1 allocation using random permuted blocks of varying size (for unpredictable allocation sequence [20]), prepared by the Nottingham Clinical Trials Unit (NCTU) statistician and held on a secure server. To obtain balanced groups on severity, block randomisation will be used to stratify participants into two groups according to overground gait speed (Severe group: <0.4 m/s; Moderate group: between 0.4 m/s and 0.8 m/s [19]). Participants will be randomly assigned to one of three treatment groups by means of a web-based randomisation system accessed by a researcher after obtaining consent and performing baseline assessments. Participants and therapists will not be blinded to the intervention allocation. The treating therapist will be notified of treatment allocation directly from the NCTU by email. To preserve allocation concealment [20], the independent assessor responsible for collecting the outcome measures will receive only blinded confirmation of randomisation. The assessor will record a guess of participants’ group allocation for later examination of the success of blinding.

**Figure 1:**
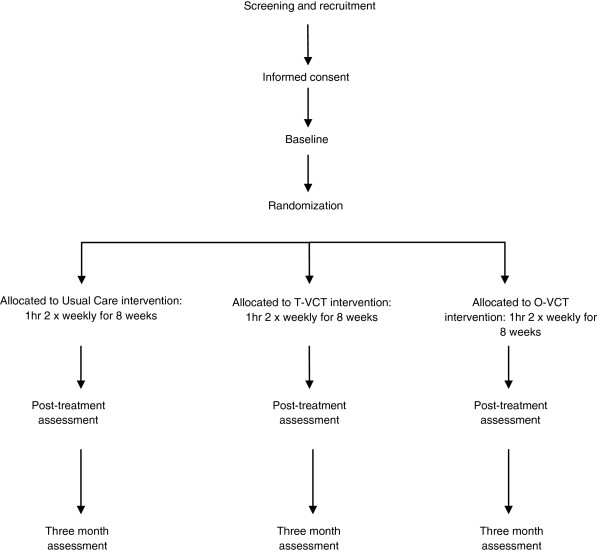
Trial design flow diagram.

The sequence from screening, enrolment (provision of written informed consent) and randomisation is represented in a flow diagram Figure 1.

### Participants

Combined inclusion and exclusion criteria are as follows:

•Community dwelling post-stroke participants over 18 years of age identified at discharge from inpatient acute wards and at referral to community and outpatient services.

•Able to provide informed consent and eligible to receive formal gait rehabilitation as indicated by:

○ Gait speed <0.8m/s corresponding with limited community ambulation ability [19];

○ Residual paresis in the lower limb (Fugl-Meyer [21] lower limb score less than 34);

○ A premorbid (retrospective) modified Rankin Scale [22] score of greater than 3;

○ Without gait deficits attributable to non-stroke pathology.

•Able to take part in practice of walking including visual cues as indicated by:

○ Walking with minimal assistance, functional ambulation category [23] of 3 or more;

○ Ability to follow a three-step command (as assessed by modified mini-mental status exam);

○ Without visual impairments preventing use of visual cue training.

•Medically stable to take part in walking rehabilitation as indicated by:

○ Without concurrent progressive neurologic disorder, acute coronary syndrome, severe heart failure, confirmed or suspected lower-limb fracture preventing mobilization;

○ Not requiring palliative care.

Patient characteristics including stroke date and lesion location, demographics, Sheffield screening test [24], mini mental state examination [25], pre-morbid modified Rankin Scale [22], and visual attention (Apple Test) [26] will be recorded.

### Interventions

This study will contrast the feasibility and potential efficacy of two forms of VCT to usual care walking (UC) rehabilitation; overground VCT walking (O-VCT) and treadmill-based VCT (T-VCT). In T-VCT, a force-instrumented treadmill (CMill, Forcelink, NL, USA) will be used to illuminate footfall targets at specified locations 2–3 steps in advance, in line with current knowledge of gaze behaviour during locomotion [27] according to gait event detection of the ongoing gait cycle [28]. In O-VCT, therapists will manually place footfall target at specified locations, according to the baseline gait assessment, along an overground walkway.

Both VCT interventions are designed to target the essential control and functional requirements of walking, namely (1) speed, (2) symmetry (equality of step length), and (3) adaptability to behavioural goals of the participant and environmental constraints, including abilities for turning and shortening, lengthening or narrowing (e.g., tandem walking) of steps [29]. The potential efficacy, feasibility and acceptability of both O-VCT and T-VCT treatment modalities are being investigated because some studies [7,30] have indicated support for mechanically aided rehabilitation approaches due to the capacity to deliver high dosage and high intensity training protocols incorporating motor learning and motor control theoretical perspectives. However, the efficacy of electromechanically aided walking practice has not been established and so they are not often offered as part of current practice [31-34].

There will be three treatment arms all of which share the same frequency, duration and intensity in terms of encouraging therapists to maintain equal session durations and same intensity of continuous walking. Participants will receive walking practice for one hour, 2 times per week, for 8 weeks duration. The target exercise capacity is 20 minutes of continuous, independent walking, with symmetrical step length. A resource usage log will record all other aspects of therapy such as occupational therapy, for which participants are referred and will continue to receive, irrespective of treatment allocation.

O-VCT and UC will be delivered in four participating hospital settings embedded within current service provision. Only one specialized treadmill (CMill, Forcelink, NL, USA) for T-VCT is available to the study and so the feasibility of this treatment is being assessed through treatment delivery at one regional treatment site (within a mean 11 mile radius of participating NHS sites delivering O-VCT and UC treatment arms) at the University of Birmingham. Patients recruited from the participating hospitals will travel to the University to receive this arm of training if they are randomised to it. This is not additional travel, but in lieu of their normal transfer to hospital for treatment. This model of a regional treatment centre is in line with the provision of other specialist rehabilitation services such as functional electrical stimulation falls efficacy scale and gait assessment in this part of the UK.

Treating therapists will receive training and a detailed treatment manual to promote consistency between therapists and sites. Adherence to the intervention by therapists will be assessed during their involvement in the trial by A. Wimperis and K. Hollands through video observation at weeks 2 and 6 of each therapists’ first treatment period. Further training for the therapist will be provided, if necessary, to improve compliance with treatment protocols. The involvement of different therapists and different sites promotes generalizability, providing multiple viewpoints regarding the treatment and its feasibility for delivery across different modes of service provision.

### VCT interventions (Figure 2 and Table 1)

**Figure 2:**
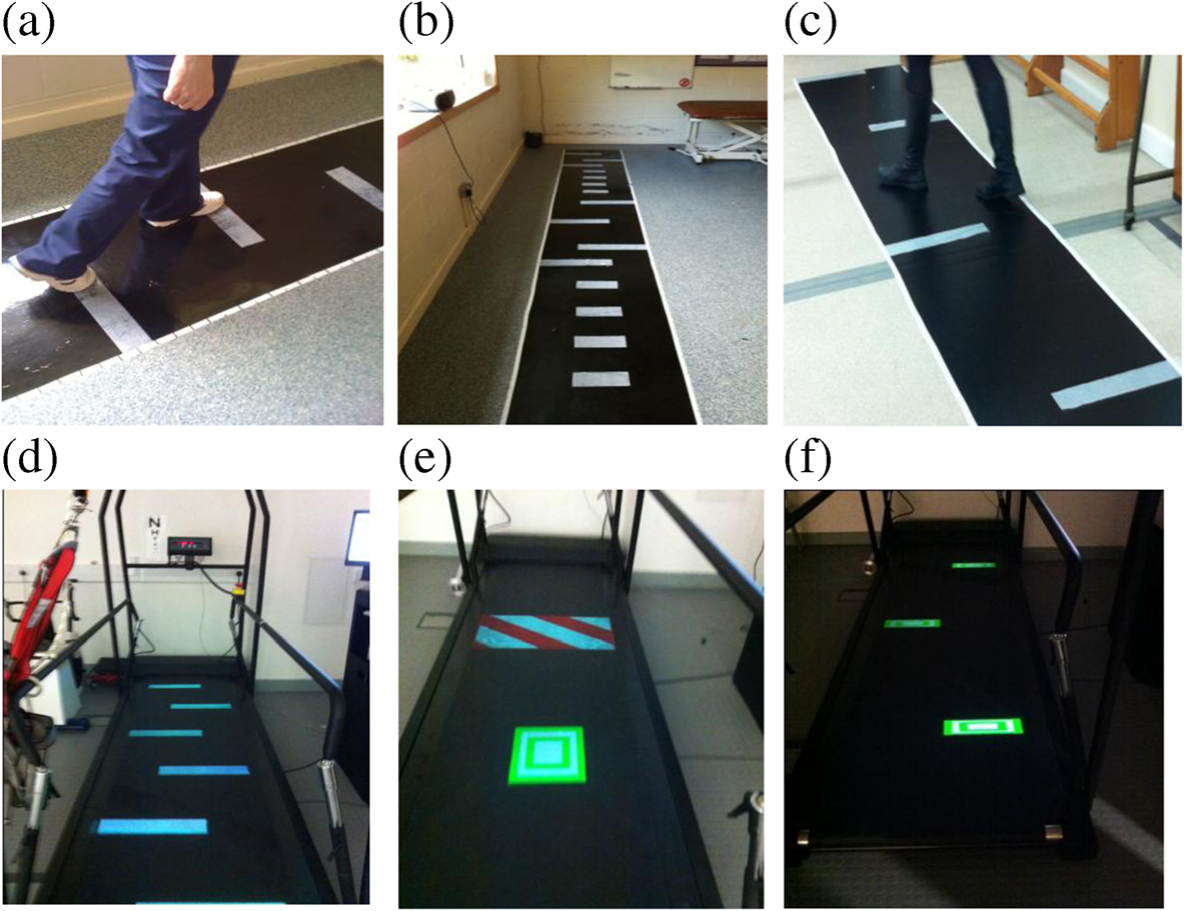
**Illustration of training target placement for O-VCT and T-VCT. (a)** O-VCT symmetry, **(b)** O-VCT adaptability, **(c)** O-VCT turning, **(d)** T-VCT symmetry, **(e)** T-VCT adaptability, **(f)** T-VCT turning.

**Table 1:** Visual cue training (VCT): treatment progression

**Progression**	**Treatment goal categories**				
**treatment phase (sessions)**	**Walking speed target**	**Symmetry target**	**Turning ability target**	**Gait adaptability/ translation to functional mobility**	**Intensity**
I (1–4)	Increasing walking speed in 10% increments, as tolerated, from baseline to the target threshold (either 0.4m/s or 0.8m/s depending on initial SSWS)	Improving symmetry of (a)step-length, (b) stance and swing phases in 10% increments, as tolerated			Four 5 min bouts of walking to total 20 min of stepping with each bout addressing one of the goals at a time
II (5–10)	Increasing walking speed in 10% increments, as tolerated, from baseline to the target threshold (either 0.4m/s or 0.8m/s depending on initial SSWS)	Improving symmetry of step-length, stance and swing phases in 10% increments, as tolerated, while maintaining new walking speed	10% improvement in turning towards ability to turn in two steps, 2 seconds in either direction while maintaining new walking speed	10% improvement in the number of failures to hit targets presented unpredictably in timing and location on both limbs while maintaining new walking speed	Increase bout duration and decrease number of bouts; however, each goal is still addressed individually in blocks of practice
III (11–16)	Practice at maintenance of walking speed over threshold and at altering speed as dictated by varying speed of presentation of footfall targets	Practice at maintenance of symmetrical stepping	Two steps, 2 seconds in either direction when turns are unpredictable	Able to alter stepping pattern to hit targets presented unpredictably in timing and location on either limb	20–30 min of sustained *good quality stepping

*Good quality stepping is defined as walking with spatial symmetry of stepping pattern and dynamic trunk control during adaptations to step length and turning.

Self selected walking speed SSWS.

#### Training of speed and symmetry

To improve symmetry we increase the shorter step length incrementally by 10% of the maximum step length. Patients are presented with stepping targets (white rectangles, 8 cm deep × 40 cm wide (which adhere to the walkway in O-VCT or are illuminated on the treadmill belt in T-VCT) along a 5 m long walkway (O-VCT), or 3 m treadmill belt (T-VCT), to which they must aim to step on. In both VCT treatment arms, stepping targets can be seen at least two steps in advance in accordance with visuo-motor control literature indicating where healthy adults typically look while walking to targets [35]. The width of the stepping targets corresponds to half of the width of the walkway, allowing for self-selected width of stepping such that medial stabilisation strategies are not constrained while patients are being challenged to alter step length and speed. The depth of the targets corresponds to the variability in step length reported in stroke patients [36]. Participants are instructed to step on the targets with any part of the foot. Thus, the depth of the targets has been selected such that they should only be missed if the error in footfall location is greater than usual variability. The location of the stepping targets is predetermined according to goals for 10% increments in improved symmetry and altered as treatment progresses to increase intensity. The location of targets is calculated and prescribed to treating clinicians by the research assessor following baseline overground gait assessment and prior to the start of training. Prescribed targets for progressing speed (beyond 0.4 m/s or 0.8 m/sfor the moderate and severely impaired, respectively) will also be provided according to 10% increments to baseline measures. Participants are allowed to use a walking aid and prescribed ankle/foot orthoses or to grasp the therapist’s hand, wall or handrail for safety. Stepping towards increasingly symmetrical targets is practiced at increasing walking speed as treatment progresses (Table 1).

#### Adaptability practice

Stepping targets are placed to elicit step adjustments similar to that required in environments with clutter or situations requiring alterations to foot-placement or direction. Targets are located along the walkway/treadmill belt to elicit lengthening, shortening (±25% of baseline step lengths) and narrowing of paretic and non-paretic steps. In the T-VCT treatment arm, illuminated targets shift to elicit step alterations at varying times in the ongoing gait cycle and obstacles are presented in red and white stripes to be avoided (Figure 2e). Thus with the exception of obstacle avoidance in T-VCT and the ability to practice changes to walking in time-critical manner, the number and magnitude of each type of step alteration are the same across both VCT treatment arms.

#### Turning practice

Turning is performed by walking between targets located 1 m apart alternately on the left and to the right of the walkway/treadmill belt. Participants are instructed to ‘turn to walk between the obstacles’ in such a fashion as to slalom their way across the walkway/treadmill belt. In O-VCT, once the end of the path is reached, participants practice a 180° turn using a horizontal marker to cue foot placement according to a two-step turn seen in healthy adults, [35,37,38]. Participants then slalom their way back down the path.

Each session will consist of 5 min each of warm up and stretching, 20–30 min (plus 10 min for rests as required) overground walking practice training programme and a 5 min cool-down. Each of the components of walking ability (symmetry, adaptability and turning) is practiced in blocks at increasing speed as treatment intensity progresses. Thus, walking is practiced in accordance with current recommendations of motor learning [8,39], i.e., with many repetitions with increasing intensity, variation of parameters, in response to external demands and using implicitly known (visuo-motor) control of the gait cycle [35].

Treatment will progress in phases layering practice of walking speed, symmetry, adaptability and turning in bouts of practice, as detailed in Table 1. Participants will continue to the next treatment phase even in cases where goals have not been met.

Walking speed in the O-VCT arm will be monitored and progressed by timing walks, with therapists’ use of a stopwatch, and feedback to the participant. T-VCT treatment will be delivered by an experienced, HPC registered research therapist. The CMill uses an assessment of each footfall to determine the timing and location of visual cues projected as light targets shone 2–3 steps ahead on the treadmill. The location of visual cues and progression of treatment will therefore be pre-programmed according to baseline gait parameters in the same manner as for the O-VCT treatment. A safety harness is worn at all times during T-VCT treatment.

### Usual care

The purposes of the UC group are to provide (1) a task-specific-based intervention that does not include use of visual cues specifically designed to influence quality or adaptability of gait; (2) an equal number of interactions and time spent with a physical therapist to minimize any potential for bias due to differential exposure and minimize the risk for differential loss to follow-up; and (3) a credible training program so that the participants would consider themselves involved in meaningful therapy activity. UC is standard NHS physiotherapy, broadly defined as a task-specific-based intervention that may involve walking overground or on a treadmill; components of gait (such as weight shifting or initiation); exercises aimed at improving upper or lower extremity strength; balance and coordination; prescription of assistive devices (such as orthotics or walking aides). UC may involve any standard equipment or objects such as cones or beanbags, which may be incorporated into walking practice for functional use, e.g., picking up. These objects will not be used specifically as visual cues for foot placement, symmetry or timing of gait, or by way of aiming to avoid or hit targets. The content of UC treatment will be captured by a treatment log for the purpose of capturing UC physiotherapy specifically used to influence walking. Therapists complete the log by ticking relevant categories for environment, aids and equipment used, activities undertaken, facilitation and feedback provided, and duration of each treatment session.

### Primary outcomes

Primary outcome measures for this early phase trial focus upon the feasibility and safety of treatment. In order to determine whether a large late phase trial is warranted, we are investigating recruitment, participation, compliance, and safety of the interventions. Outcome measures therefore include:

1. The numbers of patients willing to be recruited into both control and VCT groups.

2. The willingness of physiotherapists at each collaborating site to enrol patients, i.e., the number of potentially eligible participants referred to the study.

3. The numbers of patients who do not complete the allocated treatment, thus dropping out of the study, and the reasons for dropping out.

4. Completeness of outcome data, i.e., percentage of patients with no missing values in outcome assessments.

5. Number and type of adverse events that can be directly attributed to the project intervention.

### Secondary outcome measures

Potential for efficacy will be assessed through measures reflecting the primary aims of the intervention, i.e., speed and symmetry, turning ability, and adaptability of walking.

Primary measures of walking ability:

1. Gait speed: Proportion of participants achieving a gait speed of 0.4 m/s and 0.8 m/s, measured during a 10 m walk [40]. Perry et al. [19] have shown that these gait speed classifications correspond to walking abilities in the community, with a gait speed of <0.4 m/s for household walkers, 0.4–0.8 m/s for limited community walkers, and >0.8 m/s for community walkers. It has been demonstrated that progressing from one of these classifications to the next correlates with improvement in physical functioning and quality of life [41] and these categories also correspond with changes in the functional ambulation category, a categorical scale rating level of skill in functional ambulation [23].

2. Symmetry and turning ability: Time taken (s) and number of steps to complete a 180° turn will be measured on the GaitRite instrumented walkway. Time taken to turn will be calculated as the difference (in time) between the first footfall over a line (tape mark) on the pressure sensitive walkway delineating where to turn and the first footfall over the line on the return walk. Longer time to turn and increased number of steps to turn have all been identified as performance measures which may be indicative of difficulty turning and increased falls risk [37,42]. Additionally, spatial-temporal gait parameters will be measured during walking over the GaitRite to quantify symmetry of left and right steps. Stepping strategies during turning will be measured through gait parameters calculated by GaitRite software including step width, step length (relative to line of progression) [43], and single support time during turning steps.

3. Adaptability of gait: The number of times participants fail to hit stepping targets arranged to cue varying (baseline step length ±30% and medial) foot placements on the overground walkway (as previously described and illustrated in Figure 2). A target is classified as missed if the participant is visually observed to be unable to place the whole foot accurately on the target independently and safely (according to visual inspection). The assessor documents the number of targets missed in three consecutive passes of the walkway (a total of 48 targets including three attempts of each step adjustment on each side) as well as time taken to complete each pass of the walkway and a score for the level of supervision or assistance required.

We will further explore the relationship between gait impairments and activity level measures of independence of functional walking in the community. Therefore, the following secondary outcome measures are included:

•The timed up and go test has previously been shown to have good test-retest reliability in stroke patients [44,45]. The time taken with a stopwatch will be used to test ability to walk and turn in the context of this standardised test of everyday functional mobility.

•Fugl-Meyer assessment [21] will be used to assess changes in motor and sensory impairment.

•Berg balance scale [46,47] will be used to capture any effects of interventions on balance.

•Overall independence of mobility in the community setting will be rated using the functional ambulation category [48].

•Falls efficacy scale [49] will be used to assess changes in confidence to walk without falling, which may be expected as a result of practice of adaptable walking.

•SF-12 [50,51]. This is a short-form health survey with only 12 questions. It yields an 8-scale profile of functional health and well-being scores, including physical functioning, and social, emotional, mental and general health, and has been included to measure effects on broader quality of life (Table 2).

**Table 2:** Assessment schedule and measures

**Outcome measure**	**Clinical status**	**baseline**	**Post-intervention (8 weeks post-randomisation)**	**Follow-up (3 months post-randomisation)**
Stroke date and lesion location	X			
Demographics	X			
Sheffield screening test [34]	X			
Mini mental state examination [35]	X			
Premorbid modified Rankin Scale [19]	X			
Visual attention – Apple test [36]	X			
Gait assessment (symmetry measures)		X	X	X
Gait speed (10 m walk test)		X	X	X
Gait adaptability (number of targets missed)		X	X	X
Timed up and go [37]		X	X	X
Fugl-Meyer [18] motor assessment (lower limb extremity)		X	X	X
Berg balance scale [38-40]		X	X	X
Functional ambulation classification [41]		X	X	X
Short form 12 (SF-12) [42,43]		X	X	X
Falls efficacy scale [44]		X	X	X

### Economic evaluation

The purpose of the economic evaluation in the pilot trial is to identify all the relevant health and social care costs, and pilot methods for collecting cost data (data collection forms, questionnaires). This will provide initial cost information and enable effective resource use and cost data collection systems to be determined for a subsequent definitive trial.

#### Data collection

Details of all travel to and equipment used for patient therapy will be recorded and costs established. Therapist time spent with the patient and location of therapy will be recorded in order to calculate the cost of an individual session. Any additional stroke rehabilitation-specific primary and secondary care and social services resource use information will be collected in a self-report log from each patient. Quality of life will also be measured using responses to the SF-12 at baseline, 8 weeks and 3 months.

#### Analysis

Unit costs will be applied to all items of resource use, and health and social care costs per patient will be estimated. Responses to the SF-12 will be converted to SF-6D scores, allowing the calculation of quality adjusted life years (QALYs) per patient over the 3-month period. A cost-consequence analysis will be undertaken presenting all costs and outcomes in a disaggregated form for each trial arm. Within the economic evaluation, alongside a larger trial, a cost-effectiveness analysis and cost-utility analysis are proposed to determine the cost per unit reduction in impairment, and cost per QALY gained (using the SF-6D derived from SF-12 responses).

### Assessment of safety and adverse event monitoring

The risk of serious or adverse events from taking part in the study is considered low; however, as with conventional gait rehabilitation, there is a small possibility of injury as a result of a fall. No special safety assessments are planned. Clinicians will be advised that participant safety is paramount. Walking will be practiced within limits considered by the therapist to be safe at the time and targets for treatment progression will only be used as a guideline. In addition to the compliance of standard NHS reporting procedures, adverse events, including falls, will be reported immediately to the study coordinating centre via email using an adverse event form and then quarterly to the Trial Steering Committee (TSC) and Data Monitoring and Ethics Committee (DMEC). Adverse events will be reviewed immediately by the treating therapist and the research therapist to determine the severity, cause and likelihood of recurrence. Training will be discontinued if the treating therapist or research therapist deems continuation unsafe.

### Ethical approvals and data monitoring

Ethical approval has been granted by the NRES Committee West Midlands (11/WM/0167). R&D Governance approval is provided by the University of Salford; BBC CLRN RM&G Consortium Trusts (284.74472.P); Heart of England NHS Foundation Trust (2011007SKE); South Warwickshire NHS Foundation Trust (74472); and Sandwell and West Birmingham Hospitals Trust (11STR07). The trial is registered on the ClinicalTrials.gov database (NCT01600391) and adopted by the Stroke Research Network UKCRN (ID11147). A combined TSC and independent DMEC will be used to monitor the trial conduct. The grant holding team meet to review project management on a quarterly basis with day-to-day management overseen by the chief investigator CI.

Data quality is ensured both through the monitoring of the TSC and DMEC, and through the engagement of data services of the NCTU. The NCTU maintains the computer-based database of case report forms and has developed and tested the validations for entering study data into the database.

Compliance to the trial protocol by participating NHS sites is ensured by provision of a treatment manual and training of all participating NHS therapists by the research therapist and assessor, as well as video observation of treatment delivery.

### Statistical analysis

This feasibility study is designed primarily to test recruitment, retention and the completeness of data that could be expected within a definitive multicentre trial, hence, there will be no formal statistical assessment of clinical efficacy. Secondary outcome measures will be summarised and mean differences between the arms will be calculated and presented with confidence intervals to determine sample sizes for a subsequent late phase trial. No interim analysis will be conducted.

Screening logs will be held centrally for each site and from which monthly recruitment rates will be accrued together with the percentage of participation refusals. The screening data will be analysed to determine characteristics of the excluded samples. The number of withdrawals before and after randomisation will be monitored by the NCTU together with a primary reason for withdrawal whenever possible. Demographic and other baseline data will be summarised by descriptive statistics (number, mean, standard deviation, median, minimum and maximum) or frequency tables, stratified by treatment.

Compliance in terms of treatment sessions attended will be summarised by descriptive statistics (number, mean, standard deviation, median, minimum and maximum) or frequency tables, stratified by treatment. The frequency, type and duration of exercises will be summarised from treatment logs recorded by physiotherapists at each session. These will be collated after the follow-up assessment to prevent unblinding. The completeness of treatment log data will then be determined. Adverse event incidents will be summarised. A telephone interview will be conducted at the end of the patients’ participation to determine patient views on the VCT training. Focus group meetings will be conducted with therapists after the study to ascertain professional opinions regarding delivery of the interventions. Patient and therapist feedback will be synthesised to determine necessary changes to the interventions.

## Discussion

Recovery of independent mobility after stroke is a major priority of rehabilitation but evidence indicates that current approaches have only modest effects on walking impairment and activity [6,7]. In accordance with the suggested need for studies in this area [7], this early phase trial will test the feasibility, safety and potential efficacy of two novel approaches which offer repetitive task-specific practice of walking in response to visually cued external demands designed to exploit implicitly known visuo-motor control of the gait cycle. Results will indicate potential response (e.g., a confidence interval indicating if, and which of the VCT interventions has the potential to be superior to the UC) to walking practice incorporating visual cues, stepping adaptability and turning practice in contrast to UC in community-dwelling stroke survivors.

For clinicians, this research will help to define an evidence-based protocol for VCT within routine practice, which is targeted towards increased speed, improved spatial symmetry and dynamic gait control during adaptations to step length and turning. It is anticipated that the resulting improvements to functional gait will reduce dependence upon carers and promote physical activity and social participation for people with stroke, and further, that reduced health-care costs will reflect fewer falls.

## Trial status

Recruitment began in June 2012. To date 364 stroke patients have been screened for eligibility, 32 potentially eligible participants have been approached for consent, and 8 have declined. Out of the remaining 24 eligible participants, 16 have provided consent and 8 consents are pending; 11 participants have been randomised and 5 are awaiting baseline assessment prior to randomisation.

## Abbreviations

DMEC: Data monitoring and ethics committee; NCTU: Nottingham clinical trials unit; O-VCT: Overground VCT; TSC: Trial steering committee; T-VCT: Treadmill-based VCT; UC: Usual care; VCT: Visual cue training.

## Competing interests

The authors declare they have no competing interests.

## Authors’ contribution

KH conceptualized the study, contributed to the design and the procurement of funding, developed procedures for implementing the protocol, oversees coordination of the trial and helped draft the manuscript. PvV conceptualized the study, contributed to the design and the procurement of funding and developed procedures for implementing the protocol. CS contributed to the design and the procurement of funding and developed procedures for implementing the protocol. DW contributed to the design and the procurement of funding, and developed the database for randomisation and performed statistical analysis. SJ developed procedures for implementing the protocol and performed health economic analyses. AWing contributed to the design and the procurement of funding and developed procedures for implementing the protocol. AWimperis contributed to the design and the procurement of funding and developed procedures for implementing the protocol. TP developed procedures for implementing the protocol, conducted all blinded assessments, participated in the coordination of the trial and helped to draft the manuscript. All authors contributed to and have checked the final manuscript. All authors read and approved the final manuscript.
